# Relationship between diabetes mellitus and atrial fibrillation prevalence in the Polish population: a report from the Non-invasive Monitoring for Early Detection of Atrial Fibrillation (NOMED-AF) prospective cross-sectional observational study

**DOI:** 10.1186/s12933-021-01318-2

**Published:** 2021-06-24

**Authors:** Jakub Gumprecht, Gregory Y. H. Lip, Adam Sokal, Beata Średniawa, Katarzyna Mitręga, Jakub Stokwiszewski, Łukasz Wierucki, Aleksandra Rajca, Marcin Rutkowski, Tomasz Zdrojewski, Tomasz Grodzicki, Jarosław Kaźmierczak, Grzegorz Opolski, Zbigniew Kalarus

**Affiliations:** 1 Liverpool Centre for Cardiovascular Science, University of Liverpool and Liverpool Heart & Chest Hospital, Liverpool, UK; 2 Aalborg Thrombosis Research Unit, Department of Clinical Medicine, Aalborg University, Aalborg, Denmark; 3 DMS in Zabrze, Department of Cardiology, Medical University of Silesia, Katowice, Poland; 4 Department of Cardiology, Silesian Centre for Heart Diseases, Zabrze, Poland; 5 National Institute of Hygiene, Warsaw, Poland; 6 Department of Internal Medicine and Gerontology, Jagiellonian University Medical College, Kraków, Poland; 7 Department of Cardiology, Pomeranian Medical University, Szczecin, Poland; 8 First Chair and Department of Cardiology, Medical University of Warsaw, Warsaw, Poland; 9 Department of Preventive Medicine and Education, Medical University of Gdansk, Gdańsk, Poland; 10 Silesian Park of Medical Technology Kardio-Med Silesia in Zabrze, Zabrze, Poland

**Keywords:** Atrial fibrillation, Diabetes mellitus, Epidemiology, Prevalence, Long-term monitoring

## Abstract

**Background:**

The global burden of atrial fibrillation (AF) and diabetes mellitus (DM) is constantly rising, leading to an increasing healthcare burden of stroke. AF often remains undiagnosed due to the occurrence in an asymptomatic, silent form, i.e., silent AF (SAF). The study aims to evaluate the relationships between DM and AF prevalence using a mobile long-term continuous ECG telemonitoring vest in a representative Polish and European population ≥ 65 years for detection of AF, symptomatic or silent.

**Methods:**

A representative sample of 3014 participants from the cross-sectional NOMED-AF study was enrolled in the analyses (mean age 77.5, 49.1% female): 881 (29.2%) were diagnosed with DM. AF was screened using a telemonitoring vest for a mean of 21.9 ± 9.1days.

**Results:**

Overall, AF was reported in 680 (22.6%) of the whole study population. AF prevalence was higher among subjects with concomitant DM (DM+) versus those without DM (DM−) [25%, 95% CI 22.5-27.8% vs 17%; 95% CI 15.4–18.5% respectively, p < 0.001]. DM patients were commonly associated with SAF [9%; 95% CI 7.9–11.4 vs 7%; 95% CI 5.6–7.5 respectively, p < 0.001], and persistent/permanent AF [12.2%; 95% CI 10.3–14.3 vs 6.9%; 95% CI 5.9–8.1 respectively, p < 0.001] compared to subjects without DM. The prolonged screening was associated with a higher percentage of newly established AF diagnosis in DM+ vs DM− patients (5% vs 4.5% respectively, p < 0.001). In addition to shared risk factors, DM+ subjects were associated with different AF and SAF independent risk factors compared to DM− individuals, including thyroid disease, peripheral/systemic thromboembolism, hypertension, physical activity and prior percutaneous coronary intervention/coronary artery bypass graft surgery.

**Conclusions:**

AF affects 1 out of 4 subjects with concomitant DM. The higher prevalence of AF and SAF among DM subjects than those without DM highlights the necessity of active AF screening specific AF risk factors assessment amongst the diabetic population.

*Trial registration*: NCT03243474

**Supplementary Information:**

The online version contains supplementary material available at 10.1186/s12933-021-01318-2.

## Background

Atrial fibrillation (AF) is the most prevalent cardiac arrhythmia worldwide [1–3]. AF is associated with substantially impaired quality of life by increasing the risk of stroke, thromboembolism, heart failure, dementia and all-cause mortality, thus constituting the public health priority of utmost importance [2, 4–7]. The global burden of this arrhythmia is rapidly increasing due to the widespread ageing of the population and related occurrence of numerous comorbidities.

Nonetheless, the AF prevalence in many prior studies is likely to be underestimated due to many patients with silent AF (SAF), an asymptomatic form of arrhythmia, which remains undiagnosed. For that reason, although comprehensive and holistic AF management [8] is crucial, the active screening and early detection of the arrhythmia poses a real challenge.

The likelihood of establishing the diagnosis increases along with the monitoring timespan [9]. These emphasize the necessity of actively searching for AF, preferably taking advantage of non-invasive wearable ECG-monitoring devices, to maintain the balance between efficiency and compliance in predicting rhythm disturbances [10, 11]. In a cross-sectional epidemiological study, the Non-invasive Monitoring for Early Detection of Atrial Fibrillation (NOMED-AF)**,** we investigated the AF prevalence in the European population aged ≥ 65 using long-term continuous monitoring [12].

One of the common aetiological factors for incident AF is diabetes mellitus (DM) [13]. Indeed, DM is the most predominant metabolic disorder in the general population, affecting 451 million people in 2017 with an increase projected to 693 million by 2045 [14]. Not only is diabetes a well-known major risk factor for AF development, but it is also associated with stroke, thromboembolism and is a cornerstone of metabolic syndrome, which has an adverse impact on the overall prognosis [15, 16]. Multiple studies have reported the association between DM and substantially increased risk of AF incidence [17–22].

In this ancillary analysis to NOMED-AF, we aimed to evaluate the relationships between DM and AF prevalence using a mobile long-term continuous ECG telemonitoring vest in a representative Polish population ≥ 65 years for detection of both symptomatic or silent AF.

## Methods

The study was conducted as a sub-analysis of Non-invasive Monitoring for Early Detection of Atrial Fibrillation (NOMED-AF) study, a cross-sectional observational study aiming to evaluate the AF prevalence and its associated comorbidities in the Polish population. The detailed study protocol has been previously described [12]. The study used a long-term wearable non-invasive ECG monitoring system linked with an online platform for data analysis and storage.

The enrolment period was between March 15th, 2017 and March 10th, 2018. The trial schedule comprises multistage, stratified and clustered population sampling, during which the representative Polish population ≥ 65 was stratified by province and place of residence. The procedure is thoroughly described in the Additional file 1: appendix. Briefly, the whole country territory was stratified into 59 geographical strata. After that, the regions from each stratum (villages, towns, cities) were randomly selected by the proportional probability, the study participants from the previously chosen areas were also selected at random manner, based on the personal identity number. A similar number of men and women in each 5-year age group were designated. Therefore, we achieved the oversampling of older age groups. We did the process to assure that the size of the final subsample of the oldest subjects will be enough for separate analyses. The oversampling was corrected at the stage of statistical analysis with weights to get population estimates. For each of the 3000 participants, another 9 subjects living in the same cluster were drawn. These “spare” addresses were used in a predefined random order only if the address of the primarily chosen subject was incorrect or a subject refused to take part in the study.

### Data collection

Each of the study participants was interviewed at home by a trained nurse, using a standardized questionnaire. Relevant questions for the current analysis are as follows: previously diagnosed AF, symptoms and signs related to AF, symptoms of other cardiovascular diseases, presence of concomitant diabetes mellitus or chronic kidney disease. There were also collected data needed to calculate CHA_2_DS_2_-VASc score.

Moreover, height and weight were taken from each participant. Blood pressure was measured during two separate visits at home, using validated automated oscillometric devices. Urine and fasting blood samples were collected and processed in the central laboratory.

### Long term ECG monitoring

Thirty-day, surface, 2-lead ECG recording was attempted in each of the study subjects, including respondents with already established AF diagnosis. The dedicated ECG monitoring system was developed and manufactured by Comarch Healthcare (Krakow, Poland) specifically for the purpose of the current study. The system consisted of a vest equipped with ECG leads, two exchangeable recorders and a docking station allowing to charge recorders and transmit data, while another recorder was at the same time connected to vest and recording. ECG data was transmitted to a central database with the use of GSM technology.

The ECG recording was screened automatically for AF and atrial flutter episodes lasting longer than 30 s, using software developed and validated, especially for the purpose of the study. Episodes of atrial fibrillation/atrial flutter lasting longer than 30 s were automatically detected by AF detection algorithms of the analytical platform. Finally, each of the automatically detected episodes was reviewed by trained cardiologists.

### Outcomes

The presence of AF was established based on the patient’s medical records assessed for all subjects by the qualified study nurse on-site, confirmed by ECG record/monitoring (all participants had long term ECG monitoring). All of the newly diagnosed AF cases (not previously detected) were established based on up to 30 days of surface ECG monitoring for episodes of AF lasting 30 s or longer. Newly diagnosed AF was defined as AF found in patients without previous history of this arrhythmia in available medical documentation. In this paper, the term AF refers both to atrial fibrillation and/or atrial flutter.

Patients were diagnosed with paroxysmal AF if the duration of the recorded longest arrhythmia event was shorter than 7 days. All other cases were considered as persistent/permanent. Because it is not always possible to distinguish precisely between persistent and permanent AF using patients’ medical documentation, we analyzed both as one group.

DM type 2 diagnosis was established in line with the American Diabetes Association [23] and European Association for the Study of Diabetes [24]. Guidelines if the haemoglobin A1c (HbA1c) measured by HPLC was ≥ 6.5% or if the patient was aware of diabetes and a glucose-lowering treatment was applied. Physical activity threshold was defined as exercise at least > 30 min ≥ 3 times a week.

The studied cohort was divided into two study groups based on DM presence: DM (+) group—participants with concomitant DM; and DM (−) group—subjects without DM. AF prevalence was also analyzed in correlation to age and gender. The detailed baseline characteristics were described for both—NOMED and Polish population, while all other analyses were weighted and reported only for the Polish population. None of the study participants was reported with DM type 1, hence the analyses comprise only individuals diagnosed with type 2 diabetes.

Signed, informed consent was obtained from each eligible participant of the trial in accordance with protocol regulations approved by the local review boards governing research involving human subjects and local bioethical committee (26/2015), and the Declaration of Helsinki. The trial was registered on clinicaltrials.gov (NCT03243474).

### Statistical analysis

Continuous variables were presented as mean and standard deviation (SD). Categorical variables were depicted as counts and percentages, analyzed by chi-squared test. National estimation, i.e., the frequency of comorbidities prevalence, average values for age, BMI etc., were analyzed on weighted data. The estimations were calculated so that the sample proportions were stratified by sex, age and city class were the same as in the Polish population. 95% Confidence intervals were determined, including the complex sampling scheme and were used to express the significance of differences between specific categories. Fisher’s exact test was performed to compare differences between individual age categories. A logistic regression analysis was conducted to obtain the risk changes relative to age and sex. A multiple logistic regression analysis was conducted to obtain independent risk factors of AF and SAF in DM+ and DM- populations. The independent variable was 5-year age groups and gender. A two-sided p-value  < 0.05 was considered to be statistically significant.

Oversampling of elderly age groups was addressed by using weights, which corrected the age and sex structure of the sample to the structure of the Polish population. Statistical analyses accounted for complex survey design. Prevalence and 95% confidence intervals (95% CI) were reported.

Finally, for each patient with paroxysmal AF, the number of hours of ECG monitoring before the first recorded AF event (lasting at least 30 s) was assessed. Based on these data, the relationship between the duration of ECG monitoring and the number of AF cases was examined.

## Results

A representative sample of 3014 participants of the NOMED-AF study was eligible and enrolled in the analysis. From the study population, 881 (29.2%) people were diagnosed with DM (DM+ group). Subjects with concomitant diabetes were less likely to be female (57%; 95% CI 53.5–60.4 vs 62%; 95% CI 60.3–63.9%, p = 0.009, respectively), had higher BMI index (30.35 ± 4.98 vs 27.5 ± 4.63, p < 0.001, respectively) and more comorbidities including hypertension, heart failure, chronic kidney disease, coronary artery disease and stroke (all p < 0.001). Moreover, DM subjects had higher stroke risk according to the CHA_2_DS_2_-VASc score (5.4 ± 1.3 vs 3.8 ± 1.3 pt.; p < 0.001, respectively). Detailed baseline characteristics of the analyzed population are reported in Table 1.

**Table 1:** Baseline characteristics of NOMED-AF and Polish population

Clinical characteristics	DM−		DM+		p
	NOMED-AF population	Polish population	NOMED-AF population	Polish population	
	n [%]	% (95% CI)	n [%]	% (95% CI)	
Age overall	77.5 ± 8.06	74.5 ± 7.59	77.6 ± 7.54	75.1 ± 7.2	0.076
65–69 years	430 [20]	35 (32.8–36.9)	142 [16]	27 (23.6–30.9)	< **0.001**
70–74 years	435 [20]	22 (20.3–23.9)	197 [22]	26 (23.1–29.1)	**0.020**
75–79 years	396 [19]	16 (15.1–17.5)	189 [21]	20 (17.4–22.1)	**0.027**
80–84 years	361 [17]	13 (12.1–14.7)	168 [19]	15 (13.1–17.7)	0.166
85–89 years	324 [15]	9 (8.3–9.7)	118 [13]	8 (6.6–10)	0.458
≥ 90 years	187 [9]	5 (4.2–5)	67 [8]	4 (3.2–4.7)	0.434
Female	1070 [50]	62 (60.3-63.9)	409 [46]	57 (53.5–60.4)	**0.009**
BMI (kg/m^2^)	27.33 ± 4.560	27.50 ± 4.625	29.77 ± 4.949	30.35 ± 4.981	< **0.001**
Hypertension	1623 [77]	76 (74–77.8)	810 [92]	93 (91.8–94.7)	< **0.001**
Heart failure	431 [20]	16 (14.2–17.2)	242 [28]	25 (22.5–28.3)	< **0.001**
Chronic kidney disease (eGFR < 60ml/min)	524 [29]	24 (21.8–25.4)	285 [37]	32 (29.2–35.9)	< **0.001**
Chronic kidney disease (TOTAL)	641 [31]	24 (22.6–26)	364 [42]	37 (33.5–39.9)	< **0.001**
Haemodialysis	3[2]	1 (0.4–2.8)	5 [5]	3 (1–7.4)	0.458
Stroke	165 [8]	7 (5.–7.8)	118 [13]	13 (10.8–15)	< **0.001**
Ischemic cerebral stroke	124 [6]	5 (4.1–5.8)	82 [9]	9 (7.1–10.3)	< **0.001**
Intracranial haemorrhage	12 [1]	1 (0.3-1)	4 [0.5]	1 (0.2-2)	0.634
Unclassified stroke	29 [1]	1 (1-1.8)	32 [4]	3 (2.4-4.9)	< **0.001**
TIA	121 [6]	5 (3.8-5.4)	65 [7]	7 (5.4-9.2)	**0.006**
Coronary heart disease	392 [19]	16 (14.9–18.3)	274 [31]	29 (26.5–32.6)	< **0.001**
Myocardial infarction	253 [12]	10 (9.2–11.7)	193 [22]	21 (18.7–24.3)	< **0.001**
Peripheral artery disease	268 [13]	11 (9.5–11.9)	147 [17]	15 (12.7–17.1)	< **0.001**
CHA_2_DS_2_VASc (points)	3.60 ± 1.416	3.44 ± 1.384	5.08 ± 1.400	4.97 ± 1.414	< **0.001**
CHA_2_DS_2_VASc in AF patients	4.12 ± 1.542	3.97 ± 1.591	5.49 ± 1.372	5.47 ± 1.444	< **0.001**
CHA_2_DS_2_VASc in SAF patients	3.87 ± 1.543	3.74 ± 1.591	5.56 ± 1.450	5.57 ± 1.531	< **0.001**

AF: atrial fibrillation; BMS: body mass index; CHA_2_DS_2_-VASc: stroke risk scale (congestive heart failure, hypertension, age > 75, diabetes, stroke, vascular disease, age 65–74, sex); eGFR: estimated glomerular filtration rate; TIA: transient ischemic attack; SAF: silent atrial fibrillation

Bold value indicates in the table are considered to be statistically significant (p < 0.05)

The mean ECG monitoring interval in the studied population was 20.07 ± 8.98 days, while the mean monitoring timespan among the DM+ group was 20.31 ± 8.97 days and in DM− group, 19.97 ± 9.01 days. The mean time to detect any first AF episode was 7.25 ± 7.79 days, and for the first episode of SAF, 8.48 ± 8.29 days.

**Fig. 1:**
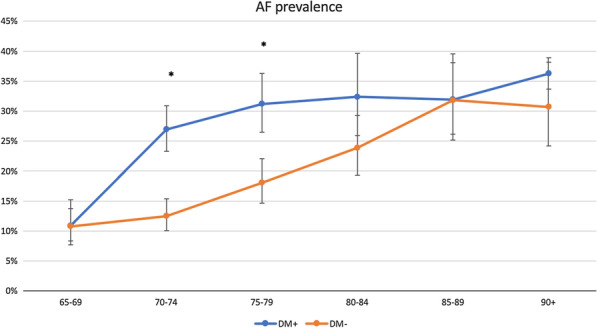
Prevalence of AF in the Polish population with (DM+) and without (DM−) concomitant diabetes mellitus in correlation to age. *p < 0.001 between DM+ and DM− study groups

**Fig. 2:**
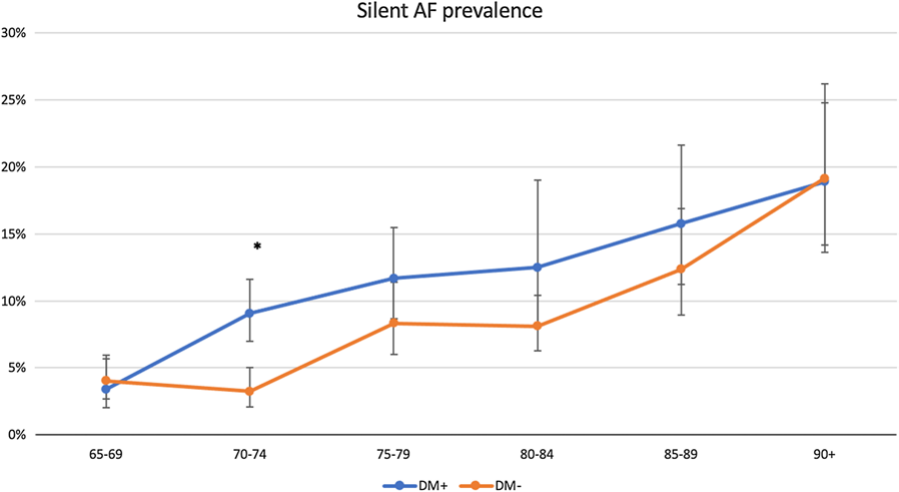
Prevalence of asymptomatic, silent atrial fibrillation (SAF) in the Polish population with (DM+) and without (DM-) concomitant diabetes mellitus in correlation to age. *p < 0.001 between DM+ and DM− study groups

Overall, AF was identified in 22.6% (n = 680) of the overall study population. The analyses, conducted with the use of data weighted for the Polish population, indicated significantly greater AF prevalence among participants diagnosed with DM compared to those without DM (25%; 95% CI 22.5–27.8% vs 17%; 95% CI 15.4–18.5% respectively, p < 0.001) (Fig. 1). DM patients were commonly associated with SAF (9%; 95% CI 7.9–11.4 vs 7%; 95% CI 5.6–7.5 respectively, p < 0.001) (Fig. 2).

Prolonged screening for AF was associated with more newly established AF diagnoses in participants with concomitant DM compared to those without DM (5% vs 4.5% respectively, p < 0.001). Also, DM+ patients had a greater prevalence of persistent or sustained AF than those in the DM− group (12.2%; 95% CI 10.3–14.3 vs 6.9%; 95% CI 5.9–8.1 respectively, p < 0.001). The arrhythmia classification is described in Table 2.

**Table 2:** AF type in NOMED-AF and Polish population

AF type	DM−			DM+			P
	NOMED-AF population		Polish population	NOMED-AF population		Polish population	
	N	%	%	N	%	%	
Total AF prevalence	427	20.0	17 (15.4–18.5)	253	28.7	25 (22.5–27.8)	< **0.001**
AF detected during the ECG monitoring	320	15.0	12 (10.9–13.7)	195	22.1	19 (16.3–21.2)	< **0.001**
AF de novo	96	4.5	4 (3.4–5)	44	5.0	4 (3.2–5.3)	< **0.001**
Paroxysmal AF	226	10.6	10 (8.7–11.4)	124	14.1	13 (11–15)	< **0.001**
Persistent or sustained AF	201	9.4	6.9 (5.9–8.1)	129	14.6	12.2 (10.3–14.3)	< **0.001**
Silent AF	181	8.5	7 (5.6–7.5)	98	11.1	9 (7.9–11.4)	**0.004**
AF silent de novo	70	3.3	3 (2.2–3.6)	35	4.0	3 (2.5–4.5)	0.389
AF or/and AFl	34	1.6	1 (0.8-1.6)	25	2.8	3 (2–4.3)	< **0.001**

AF: atrial fibrillation; AFl: atrial flutter; ECG: electrocardiography

Bold value indicates in the table are considered to be statistically significant (p < 0.05)

### Age and sex

There was increasing arrhythmia prevalence along with age noticeable in both women and men. Similar trends of rising prevalence along with age are described in SAF. Arrhythmia prevalence in 5-year age ranges is summarised in Table 3. In the general population, the AF prevalence was relevantly higher in men vs women (OR 2.56, 95% CI 1.68–3.90 p < 0.001), while in the DM+ group analysis there were no significant differences in AF prevalence between males and females (OR 1.78, 95% CI 0.75–4.32, p = 0.19). Detailed analysis of odds ratios in correlation to age and gender also indicated no differences between AF prevalence in men and women with concomitant diabetes in equivalent age groups (Additional file 2: Table S2).

**Table 3:** AF prevalence in the Polish population with concomitant DM

	Overall (N = 881)				Men (N = 472)					Women (N = 409)				
	N	N AF	% (95% CI)	p	N	N AF	% (95% CI)	OR (95% CI)	p	N	N AF	% (95% CI)	OR (95% CI)	p
Overall	881	253	25 (23–27.2)		472	147	26.7 (23.9–29.6)			409	106	23.8 (21-26.9)		
[65–69]	142	17	10.9 (7.7–15.3)	Ref.	79	11	13.5 (9.9-18.1)	Ref.	Ref.	63	6	8 (3.5-17.4)	Ref.	Ref.
[70-74]	197	48	27 (23.3–30.9)	< **0.001**	97	24	23.7 (18.3–30)	1.99 (0.99–4.03)	0.055	100	24	29.4 (24.5-34.9)	4.77 (2.17–10.51)	< **0.001**
[75–79]	189	64	31.2 (26.5–36.3)	**0.002**	109	45	40 (33.8–46.5)	4.28 (2.12–8.63)	< **0.001**	80	19	24.4 (18-32.3)	3.70 (1.61–8.53)	**0.002**
[80–84]	168	58	32.4 (25.9–39.6)	**0.001**	88	33	40 (30.1–50.8)	4.29 (1.97–9.34)	< **0.001**	80	25	27.9 (20-37.5)	4.37 (1.19–10.30)	**0.001**
[85–89]	118	41	31.9 (25.2–39.6)	**0.002**	66	26	39.4 (32.9–46.4)	4.81 (1.55–11.30)	**0.005**	52	15	28.3 (19.2-39.7)	4.53 (1.77–11.58)	**0.002**
[90–]	67	25	36.3 (33.7–38.9)	< **0.001**	33	8	24.2 (13.3–40)	2.05 (0.24–12.50)	0.438	34	17	39.5 (34.9-44.4)	7.48 (2.63–21.29)	< **0.001**

CI: confidence interval; OR: odds ratio

Bold value indicates in the table are considered to be statistically significant (p < 0.05)

### Comorbidities

When compared to those without DM, participants with AF and DM+ had a higher prevalence of following comorbidities: acute coronary syndrome (24% ± 3% vs 14% ± 2%, p = 0.003), peripheral arterial disease (PAD) (20% ± 3% vs 14% ± 2%, p = 0.048) and hypertension (97% ± 1% vs 81% ± 2%, p < 0.001). Furthermore, they were less physically active (29% ± 3% vs 44% ± 3%, p < 0.001) and significantly more obese (55% ± 3% vs 31% ± 2%, p < 0.001). A comparison of concomitant diseases between participants with AF from DM+ and DM- study groups is shown in Additional file 2: Table S1.

### Multivariate analyses

Multivariate regression analysis demonstrated that independent risk factors for AF differed between patients with and without diabetes. Apart from shared risk factors in both groups, thyroid disease (OR 1.99, 95% CI 1.38–2.87, p < 0.001), peripheral or systemic thromboembolism (OR 1.92, 95% CI 1.28–2.87, p = 0.002), prior percutaneous coronary intervention (PCI) or coronary artery bypass grafting (CABG) (OR 0.23, 95% CI 0.15–0.35, p < 0.001), hypertension (OR 2.16, 95% CI 1.27–3.68, p = 0.005), physical activity (OR 0.74, 95% CI 0.57–0.96, p = 0.021) were independently associated with prevalent AF in the DM+ population, unlike in DM− group.

Similar analysis showed that thyroid disease (OR 2.22, 95% CI 1.30–3.78, p = 0.004), peripheral or systemic thromboembolism (OR 1.89, 95% CI 1.13–3.15, p = 0.015), prior PCI or CABG (OR 0.31, 95% CI 0.17–0.58, p < 0.001), hypertension (OR 2.84, 95% CI 1.26–6.38, p = 0.012) and BMI > 30 (OR 1.49, 95% CI 1.03–2.17, p = 0.036) were independent risk factors for SAF in diabetic population (Table 4).

**Table 4:** Multivariate analysis of AF and SAF risk factors in DM+ and DM– study groups

	DM+			DM−		
	OR	95% CI	p	OR	95%CI	p
*AF risk*						
Age	1.050	1.034–1.067	< **0.001**	1.043	1.024–1.061	< **0.001**
Male	1.715	1.314–2.238	< **0.001**	2.234	1.706–2.927	< **0.001**
Myocardial infarction	1.188	0.773–1.827	0.430	0.818	0.560–1.193	0.295
Coronary artery disease	1.152	0.847–1.565	0.365	1.410	1.010–1.967	**0.043**
Thyroid disease	1.989	1.377–2.874	< **0.001**	1.237	0.860–1.778	0.251
COPD	0.884	0.571–1.367	0.577	0.841	0.576–1.228	0.369
Peripheral or systemic thromboembolism	1.919	1.280–2.879	**0.002**	1.037	0.642–1.675	0.881
PAD	1.087	0.765–1.545	0.640	0.882	0.624–1.246	0.475
TIA	1.179	0.865–1.606	0.295	1.266	0.895–1.790	0.182
PCI or CABG	0.229	0.148–0.354	< **0.001**	0.706	0.453–1.101	0.124
Heart failure	3.389	2.423–4.740	< **0.001**	2.882	2.098–3.958	< **0.001**
Hypertension	2.160	1.268–3.680	**0.005**	1.179	0.865–1.607	0.297
Chronic kidney disease	0.876	0.659–1.164	0.360	1.577	1.219–2.402	**0.001**
Physical activity	0.738	0.570–0.955	**0.021**	1.204	0.895–1.620	0.219
BMI > 30	1.445	1.148–1.820	**0.002**	1.378	1.017–1.868	**0.039**
NT pro-BNP >= 125	2.185	1.630–2.930	< **0.001**	1.920	1.275–2.892	**0.002**
*SAF risk*						
Age	1.057	1.037–1.078	< **0.001**	1.065	1.038–1.092	< **0.001**
Male	2.011	1.383–2.925	< **0.001**	2.974	2.050–4.135	< **0.001**
Myocardial infarction	1.008	0.528–1.925	0.980	0.458	0.228–0.920	**0.028**
Coronary artery disease	1.067	0.671–1.696	0.783	1.137	0.732–1.767	0.567
Thyroid disease	2.218	1.303–3.776	**0.004**	0.974	0.598–1.589	0.917
COPD	1.005	0.561–1.799	0.988	0.540	0.318–0.919	**0.023**
Peripheral or systemic thromboembolism	1.887	1.131–3.150	**0.015**	1.307	0.654–2.612	0.447
PAD	1.560	0.997–2.441	**0.052**	0.786	0.474–1.304	0.350
TIA	1.537	0.980–2.411	0.061	1.446	0.919–2.277	0.111
PCI or CABG	0.314	0.172–0.575	< **0.001**	1.144	0.593–2.205	0.687
Heart failure	1.888	1.167–3.053	**0.010**	2.160	1.412–3.304	< **0.001**
Hypertension	2.839	1.264–6.377	**0.012**	0.746	0.518–1.073	0.113
Chronic kidney disease	1.388	0.922–2.089	0.115	1.438	1.028–2.012	**0.034**
Physical activity	0.809	0.547–1.197	0.287	1.138	0.823–1.574	0.434
BMI > 30	1.493	1.027–2.171	**0.036**	1.008	0.700–1.452	0.966
NT pro-BNP >= 125	2.422	1.416–4.142	**0.001**	2.369	1.250–4.489	**0.008**

BMI: body mass index; CABG: coronary artery bypass grafting; COPD: chronic obstructive pulmonary disease; CRP: c reactive protein; PAD: peripheral arterial disease; PCI: percutaneous, coronary intervention; TIA: transient ischemic attack

Bold value indicates in the table are considered to be statistically significant (p < 0.05)

## Discussion

In this prospective cross-sectional observational study, our principal findings are as follows: (i) we found a higher AF prevalence when diabetes was present; (ii) subjects with DM are more likely to have silent, asymptomatic AF; and (iii) DM patients were more commonly associated with persistent and permanent AF, and (iv) independent risk factors for AF incidence may vary in patients with concomitant DM comparing to the general population.

To the best of our knowledge, this is the first prospective study on AF prevalence in patients with DM, which, based on a comprehensive epidemiological methodology, was conducted on a randomly selected cohort. Unlike prior surveys based mainly on registries or cohort studies, the current study was based on prolonged non-invasive continuous ECG monitoring with a mean monitoring time span of almost 22 days. The data were transmitted remotely to the cardiovascular centres and analyzed by qualified medical professionals, resulting in a more accurate investigation. Hence, our novel finding is that 1 out of 4 Polish subjects aged ≥ 65 years with concomitant diabetes has AF. Also, diabetic patients are at a substantially higher risk of AF comparing to non-DM subjects.

AF prevalence has been reported in around 1–4% of the general European population [25]. The intimate association between AF and DM has been previously reported. The Framingham Heart Study demonstrated a 40% increase in the AF incidence among patients with concomitant DM [26]. A study of nearly 846 thousand patients from Veterans Health Administration Hospitals revealed a significantly higher AF prevalence in DM patients vs the control group without this metabolic disorder (14.9% vs 10.3%, p < 0.001) [15]. Similar results were also obtained by Huxley et al. in a case-control study on a cohort of over 100 thousand subjects [17]. Finally, a systematic review based on 32 studies and over 10 million participants found a 28% higher risk of developing AF among patients with diabetes [27]. Many of these studies have been based on ‘one off’ ECG recordings, and few studies have used prolonged ECG monitoring.

Furthermore, 9% of the Polish population with coexisting DM was diagnosed with asymptomatic AF. Even short runs of SAF may increase the risk of stroke and should not be ignored [28, 29]. Indeed, the vast majority of diabetes patients aged ≥ 65 would benefit from oral anticoagulation, and Chao et al. reported that the age threshold for initiating oral anticoagulation was 50 years in an AF patient with diabetes as a single risk factor [30]. Hence, long-term monitoring plays a pivotal role in stroke prevention, which is often the first arrhythmia symptom, and the whole population age ≥ 50 with concomitant DM should be actively screened for AF, even opportunistically when they attend clinic check-ups.

Nonetheless, the associations between DM and AF have been subject to debate and controversy [31]. Although the precise pathophysiological and clinical mechanisms are still not completely understood, there seems to be a multifactorial and bidirectional influence, including atrial structural and electrical remodelling as well as autonomic regulation [32].

The Danish population-based registry studies have either pointed out that the DM occurrence did not elevate the risk of AF incidence or that the association between AF and DM was only evident among the obese [33, 34]. Furthermore, the impact of sex on incident AF also seems to be unclear [19, 35]. In our study among DM patients, there was no significant influence of sex on AF prevalence.

The current study confirms prior observations referring to a higher number of comorbidities in the AF population with diabetes versus those without. Although there are multiple reports investigating AF risk factors in the general population, analyses evaluating independent AF risk factors in diabetic patients are lacking. Hence, we conducted a multivariate analysis, which indicated that the risk factors for the arrhythmia incidence might differ in subjects with concomitant DM compared to the general population. In contrast to the entire population, in individuals burdened by DM, comorbidities such as hypertension, PAD, obesity, or thromboembolism seem to play a pivotal role in AF development. The results are compliant with the Swedish National Diabetes Register report, which emphasized the independent association of elevated blood pressure, increased BMI, and heart failure in AF development [36]. These outcomes underline that DM should not be treated as a separate disease entity but need to be considered a complex syndrome including hypertension, dyslipidaemia or thromboembolic complications. Therefore, relevant efforts should be undertaken in the holistic management of AF patients with DM.

### Strengths and limitations

As far as we are aware, this is the first observational and epidemiological study evaluating the AF prevalence in patients with concomitant DM using a nationwide, representative population sample. Furthermore, all visits and procedures conducted during the study were taken at the subject’s home; hence, even disabled and critically ill individuals were eligible to take part. Our study is also one of the few surveys using long term ECG monitoring and the first-ever, which enrolled randomly selected participants from the general population. These facts contribute significantly to objectivity and reduce possible bias. Furthermore, we analyzed independent AF risk factors in the diabetic population, which is novel and seems to be relevant in the holistic management of diabetic subjects in everyday clinical practice.

However, the study also has some limitations. Although the participants' selection was at random manner, the response rate was modest, which could possibly influence a selection bias. Nonetheless, due to the fact that presumably healthier subjects are more likely not to respond, the response rates in the study probably might be underestimated than overestimate AF prevalence. Moreover, the limited number of elderly participants with concomitant DM and AF might impact a possible bias in the analyses. Each of the study participants had blood tests taken, including fasting plasma glucose and HbA1c levels, so it is less unlikely that the population may have included a few participants with DM who remain undiagnosed. Finally, the current study is based on a nationwide representative sample from the Polish population. Therefore, the results reflect this particular population and can be directly applied only to Polish inhabitants, mainly Caucasians, who were ethnically homogenous, with universal access to healthcare.

## Conclusions

AF affects 1 out of 4 subjects with concomitant DM. The higher prevalence of AF and SAF among DM subjects compared to those without DM highlights the necessity of active AF screening and evaluation of specific AF risk factors amongst the diabetic population.

## Supplementary Information

**Additional file 1: Figure S1.** NOMED-AF – study profile.

**Additional file 2: Table S1.** Comparison of AF- patients’ comorbidities between DM- and DM+ groups. **Table S2.** Odds ratio of atrial fibrillation prevalence in Polish population with concomitant diabetes mellitus in correlation to age and gender.

## Data Availability

The datasets used and analyzed during the current study are available from the corresponding author on reasonable request.

## Abbreviations

AF: Atrial fibrillation
BMI: Body mass index
CABG: Coronary artery bypass graft surgery
CHA_2_DS_2_-VASc: Clinical prediction score for stroke risk evaluation in patients with atrial fibrillation (congestive heart failure, hypertension, age≥75, diabetes mellitus, prior stroke or thromboembolism, vascular disease, age 65–74, sex category)
DM: Diabetes mellitus
ECG: Electrocardiography
HbA1c: Haemoglobin A1c
PAD: Peripheral arterial disease
PCI: Percutaneous coronary intervention
SAF: Silent atrial fibrillation
SD: Standard deviation
95% CI: 95% confidence interval
