# Supplementary material for: Relationship between diabetes mellitus and atrial fibrillation prevalence in the Polish population: a report from the Non-invasive Monitoring for Early Detection of Atrial Fibrillation (NOMED-AF) prospective cross-sectional observational study
Source: Cardiovasc Diabetol. 2021 Jun 24;20:128. doi: 10.1186/s12933-021-01318-2 (PMC8228888; doi:10.1186/s12933-021-01318-2)
Supplement: Supplementary file 2 — Additional file 2: Table S1. Comparison of AF- patients’ comorbidities between DM- and DM+ groups. Table S2. Odds ratio of atrial fibrillation prevalence in Polish population with concomitant diabetes mellitus in correlation to age and gender. [file 12933_2021_1318_MOESM2_ESM.docx]

**Table S1**. Comparison of AF- patients’ comorbidities between DM- and DM+ groups.

|  | DM - | | | DM + | | p |
| --- | --- | --- | --- | --- | --- | --- |
| AF+ | N | % | N | % | N |  |
| Myocardial infarction | 363 | 14 ± 2 | 213 | 24 ± 3 | 576 | **0.003** |
| Coronary artery disease | 359 | 28 ± 2 | 213 | 35 ± 3 | 572 | 0.076 |
| Thyroid disease | 363 | 16 ± 2 | 213 | 21 ± 3 | 576 | 0.146 |
| COPD | 363 | 12 ± 2 | 213 | 13 ± 2 | 576 | 0.753 |
| Peripheral or systemic thromboembolism | 362 | 9 ± 2 | 213 | 12 ± 2 | 575 | 0.423 |
| PAD | 364 | 14 ± 2 | 213 | 20 ± 3 | 577 | **0.048** |
| TIA | 365 | 14 ± 2 | 213 | 20 ± 3 | 578 | 0.062 |
| PCI or CABG | 362 | 12 ± 2 | 212 | 11 ± 2 | 574 | 0.831 |
| Heart Failure | 364 | 35 ± 2 | 213 | 43 ± 3 | 577 | **0.050** |
| Hypertension | 364 | 81 ± 2 | 212 | 97 ± 1 | 576 | **<0.001** |
| Chronic Kidney Disease | 348 | 40 ± 3 | 210 | 43 ± 3 | 558 | 0.457 |
| Physical activity | 365 | 44 ± 3 | 212 | 29 ± 3 | 576 | **<0.001** |
| BMI > 30 | 356 | 31 ± 2 | 211 | 55 ± 3 | 567 | **<0.001** |
| CRP > 5 | 347 | 18 ± 2 | 210 | 20 ± 3 | 557 | 0.615 |
| NT pro-BNP >=125 | 348 | 86 ± 2 | 210 | 89 ± 2 | 558 | 0.420 |

Abbreviations: BMI – body mass index, CRP – c reactive protein, COPD- chronic obstructive pulmonary, CABG – coronary artery bypass grafting, PAD – peripheral arterial disease, TIA- transient ischemic attack,

**Table S2.**  Odds ratio of atrial fibrillation prevalence in Polish population with concomitant diabetes mellitus in correlation to age and gender.

| Age*Gender correlation | OR (95% CI) | p |
| --- | --- | --- |
| [65-69] | Ref. | Ref. |
| [70-74] | 0.42 (0.15-1.20) | 0.106 |
| [75-79] | 1.16 (0.39-3.44) | 0.793 |
| [80-84] | 0.97 (0.31-3.04) | 0.953 |
| [85-89] | 0.92 (0.24-3.63) | 0.910 |
| [90-) | 0.27 (0.03-2.21) | 0.224 |

Legend: OR – odds ratio, CI- confidence interval
